# Incompatibles of Antipyrin

**Published:** 1892-10

**Authors:** 


INCOMPATIBLES OF ANTIPYRIN.—The *Pharmaceutische Centrallbatt*, No. 29, 1892, gives the following incompatibles of antipyrin which precipitate the antipyrin from a concentrated solution: 1, concentrated solution of carbolic acid; 2, tannic acid, and preparations containing it; 3, tincture of iodine; 4, the chlorides of mercury. The following substances, also, decompose antipyrin when triturated with it: 1, calomel forms with antipyrin a toxic combination; 2, antipyrin is decomposed when rubbed with beta-naphthol; 3, with chloral, antipyrin forms an oleaginous liquid; 4, with bicarbonate of sodium it disengages the odor of ether; 5, salicylate of sodium, equal parts with antipyrin, forms an oleaginous mixture.—*Medical Age.*

